# Activation of GABA_A_ Receptors in Colon Epithelium Exacerbates Acute Colitis

**DOI:** 10.3389/fimmu.2018.00987

**Published:** 2018-05-07

**Authors:** Xuelian Ma, Qian Sun, Xiaotong Sun, Dawei Chen, Chuanfei Wei, Xin Yu, Chuanyong Liu, Yanqing Li, Jingxin Li

**Affiliations:** ^1^Department of Physiology, School of Basic Medical Sciences, Shandong University, Jinan, China; ^2^Department of Anesthesiology, Qilu Hospital, Shandong University, Jinan, China; ^3^Laboratory of Medical Chemistry, GIGA-Molecular Biology of Diseases, Faculty of Medicine, University of Liège, Liège, Belgium; ^4^Centre for Stem Cell and Regenerative Medicine, Liaocheng People’s Hospital, Liaocheng, China; ^5^Department of Gastroenterology, Qilu Hospital, Shandong University, Jinan, China

**Keywords:** gamma-aminobutyric acid, ulcerative colitis, mucosal barrier, epithelial proliferation, apoptosis

## Abstract

Emerging evidence indicates that gamma-aminobutyric acid (GABA) has many beneficial effects such as ameliorating immune and inflammatory response. But, here we reported that activation of GABA_A_ receptors (GABA_A_ Rs) aggravated dextran sulfate sodium (DSS)-induced colitis, although the expression of pro-inflammatory cytokines was inhibited. By contrast, blocking of GABA_A_ Rs markedly alleviated DSS-induced colitis. Notably, GABA_A_ Rs and glutamic acid decarboxylase 65/67 were significantly increased in colon mucosa of ulcerative colitis patients and the mouse model of colitis. Further studies showed that GABA treatment resulted in an increment of serum FITC-dextran following its oral administration, a decrement of transepithelial electrical resistance, and an increment of bacterial invasion, effects which were blocked by bicuculline. In addition, GABA inhibited the expression of tight junction proteins and mucin secretion in colitis colon. GABA also decreased the expression of ki-67 and increased cleaved-caspase 3 expression in intestinal epithelia. Our data indicate that the GABA_A_ Rs activation within colon mucosa disrupts the intestinal barrier and increases the intestinal permeability which facilitates inflammatory reaction in colon. Meanwhile, the suppression effect of GABA on pro-inflammatory cytokines leads to insufficient bacteria elimination and further aggravated the bacteria invasion and inflammatory damage.

## Introduction

Gamma-aminobutyric acid (GABA) is the major inhibitory neurotransmitter in the mature central nervous system (CNS) (1, 2). It is synthesized through the decarboxylation of glutamate by the enzyme glutamic acid decarboxylase (GAD), which includes GAD 65 and GAD 67 (3) and can bind with two major types of GABA receptors, such as GABA_A_ receptor (GABA_A_ R) and GABA_B_ receptor (GABA_B_ R). GABA_A_ Rs are hetero-pentameric chloride ion channels assembled from a repertoire of 19 subunits (α_1–6_, β_1–3_, γ_1–3_, δ, ɛ, θ, π, ρ_1–3_) (2). The most common subtypes in the CNS consist of two α subunits, two β subunits, and one copy of an additional subunit (4). The GABA_B_ R is a G protein-coupled receptor (5). In addition to its well-known effects within the CNS, GABA and its receptors are present in various peripheral tissues, such as the pancreas, gastrointestinal tract, and immune cells (6, 7). Moreover, it has been reported that the immune system harbors all of the necessary constituents for GABA signaling, and GABA itself may function as a paracrine or autocrine factor (8). Recent studies have revealed that GABA and GABA_A_ R agonists protect against many autoimmune diseases such as type 1 diabetes (9), experimental autoimmune encephalomyelitis (8), collagen-induced arthritis (10, 11), and contact dermatitis (12). The mechanisms are not fully understood. Prud’homme et al. found that GABA inhibits T-cell activation *via* GABA_A_ Rs (13). Recently, the same group demonstrated that GABA also showed anti-inflammatory effects (14). GABA therapy in the streptozotocin-induced diabetes model prevented insulitis by reducing serum levels of inflammatory cytokines (15). GABA treatment also has been demonstrated to downregulate inflammatory responses in mouse models of rheumatoid arthritis and obesity (11, 16). Collating these findings reveals that GABAergic system is potentially a new target for modulating immune and inflammatory conditions.

Ulcerative colitis (UC) is a chronic inflammatory disorder and characterized by a relapsing and remitting mucosal inflammation. The symptoms include rectal bleeding, diarrhea, and abdominal pain. While the etiology of UC is not known, a dysregulation in intestinal mucosal barrier function and mucosal immune and inflammatory responses are critical factors associated with the pathogenesis of UC. The intestinal mucosal barrier forms a physical barrier to confine luminal bacteria by separating luminal bacteria from underlying immune cells (17). Once it is breached, luminal bacteria can invade the lamina propria (LP) and initiate an inflammatory response. In turn, immune cells are recruited and produce pro-inflammatory cytokines which further impair epithelial integrity, thereby creating a vicious cycle, eventually leading to unrestrained inflammatory responses (18). Therefore, blocking this inflammatory cascade is essential for the treatment of UC. Anti-TNF-α monoclonal antibody, such as infliximab, adalimumab, and golimumab, have long been used to treat moderate-to-severe UC and are effective in inducing and maintaining remission in UC (19–21). In light of the ability for the GABAergic system to directly suppress immune and inflammatory responses, we considered it important to investigate the potential role and mechanism of GABA in UC. To accomplish this goal, we examined the effects of GABA in dextran sulfate sodium (DSS)-induced mouse model of colitis and colonic tissue samples from UC patients as well as in colonic cell lines.

In contrast to that of results from previous reports on autoimmune diseases, here we found an unexpected pro-inflammatory role for the GABAergic system in UC. Specifically, our results showed an upregulation in GABAergic signaling within colonic biopsy specimens from UC patients and in the DSS-induced colitis model. The activation of GABA_A_ Rs exacerbated intestinal mucosal damage in the DSS-induced colitis model while positively promoting immunoprotective cytokines and reducing pro-inflammatory factors, as reported previously in autoimmune diseases (8, 14, 15). In parallel with these findings, was the observation that blocking of GABA_A_ Rs significantly ameliorated DSS-induced colitis, although it boosted the production of pro-inflammatory cytokines. Further studies showed that GABA treatment inhibited tight junction (TJ) proteins expression and mucin secretion in colitis colon. GABA also inhibited colonic epithelial cell proliferation and promoted its apoptosis. The results of our current investigation indicate that GABA, when binding with GABA_A_ Rs disrupts the intestinal barrier and consequently bowel bacteria invasion to then activate severe inflammatory damage in this UC model. Moreover, the suppression effect of GABA on pro-inflammatory cytokines further aggravated the bacteria invasion and inflammatory damage.

## Materials and Methods

### Animals and Treatment

Male C57BL/6 mice, 8–10 weeks old, were housed under SPF conditions. In the first series of experiments, mice were divided randomly into six groups with eight mice per group. Acute colitis was induced by administering 3% (wt/vol) DSS at a molecular weight of 36,000–50,000 Da (MP Biomedicals, Santa Ana, CA, USA) in their drinking water for 7 days *ad libitum* and euthanized on day 7. The six groups consisted of (1) vehicle-treated controls (Control), (2) DSS in drinking water (DSS), (3) DSS mice receiving a daily i.p. injection of GABA (Sigma-Aldrich, St. Louis, MO, USA) at 5 mg/kg (DSS + GABA), (4) DSS mice receiving a daily i.p. injection of muscimol (Tocris Bioscience, Ellisville, MO, USA) at 2 mg/kg (DSS + muscimol), (5) DSS + GABA mice receiving a daily i.p. injection of (+)-bicuculine (Tocris Bioscience, Ellisville, MO, USA) at 5 mg/kg (DSS + GABA + bicuculline), and (6) DSS mice receiving a daily i.p. injection of (+)-bicuculine (DSS + bicuculline). Mice were monitored daily for weight loss, stool consistency, and fecal bleeding.

A second series of experiments was conducted using the same six treatment groups as described above, but with animals being treated for only 3 days and euthanized on day 3.

Chronic colitis was induced by three cycles of a 5-day (days 1–5, 11–15, and 21–25) treatment with 1.5% DSS in drinking water followed by 5 days (days 6–10, 16–20, and 26–30) recovery on normal water. Mice were divided randomly into six groups: (1) vehicle-treated controls (Control), (2) 1.5% DSS in drinking water (DSS), (3) DSS mice receiving a daily i.p. injection of GABA at 2.5 mg/kg during each period of recovery (DSS + GABA), (4) DSS mice receiving a daily i.p. injection of muscimol at 1 mg/kg during each period of recovery (DSS + muscimol), (5) DSS + GABA mice receiving a daily i.p. injection of (+)-bicuculine at 2.5 mg/kg during each period of recovery (DSS + GABA + bicuculline), and (6) DSS mice receiving a daily i.p. injection of (+)-bicuculine at 2.5 mg/kg during each period of recovery (DSS + bicuculline). Mice were monitored daily for weight loss, stool consistency, and fecal bleeding as described in acute colitis model. On day 30, the animals were sacrificed. Blood were collected from the retrobulbar venous plexus. The entire colon was dissected, after measuring the colon length, the distal colon was fixed in 4% paraformaldehyde for the histopathological examination.

### *In Vitro* DSS Treatment

Human Caco-2 cells were kind gifts from Prof. Keli Tian, Department of Biochemistry and Molecular Biology, School of Basic Medical Sciences, Shandong University. HT-29 cells were kind gifts from Prof. Xiaohua Hou, Tongji Medical College, Huazhong University of Science and Technology. Cells were grown at 37°C in DMEM supplemented medium with 10% FBS, 100 IU/ml of penicillin, and 100 mg/ml of streptomycin (Gibco, Grand Island, NY, USA) in a humidified atmosphere of 5% CO_2_. To test the effect of GABA on DSS-treated cells, cells were grown in 12-well plates. Fully differentiated cell monolayers were then incubated with or without 2% DSS in the absence or presence of GABA (100 μM), muscimol (10 μM), or bicuculline (10 μM) for 48 h.

### Histology

Mice were euthanized at the indicated time points. Following measurement of the colon length, distal colon tissue samples were embedded in paraffin following standard histological procedures. Four-micrometer sections were stained with hematoxylin and eosin. Histological damage was assessed based on the severity of inflammation (score 0–3), depth of injury (score 0–3), damage of the crypt (score 0–4), and percent of area involved (score 0–4) according to methods previously described (22). For the severity of inflammation scores in the colon mucosa: 0 = no or rare inflammatory cells in the LP, 1 = increased numbers of inflammatory cells in the LP, 2 = inflammatory cells extending into the submucosa, and 3 = transmural extension of inflammatory cell infiltration (23). For depth of injury scores: 0 = absence of mucosal damage, 1 = discrete epithelial lesions, 2 = mucosal erosion or ulceration, and 3 = transmural damage. For crypt damage scores: 0=none, 1=less than 5% involvement, 2 = 33% damage, 3 = 66% damaged, and 4 = complete loss of entire crypt and epithelium. For the percent area involved: 1 = 1–25%, 2 = 26–50%, 3 = 51–75%, and 4 = 76–100%. Total scores represented that of the sum of individual scores. Colonic damage score was the sum of individual scores (24).

### Cytokine Levels

Colon samples were homogenized in ice-cold PBS. The supernatants were collected and stored at −80°C for enzyme-linked immunosorbent assay (ELISA). Total amount of proteins from the different samples was determined using the Bradford assay (Sigma-Aldrich, St. Louis, MO, USA). Cytokines (IL-10, IL-12, IFN-γ, and TNF-α) in the homogenates were directly measured using a commercial ELISA kit (all from Shanghai Enzyme-linked Biotechnology, Shanghai, China) according to the manufacturer’s instructions. The optical density of the colorimetric reaction was measured at 450 nm with a plate reader.

### Intestinal Permeability

Mice were euthanized at the indicated time points. Intestinal permeability was assessed using a FITC-dextran tracer (4 kDa, 0.4 mg/g body weight, Sigma-Aldrich, St. Louis, MO, USA) as described previously (25). Mice were gavaged with 0.5 ml of FITC-dextran at 4 h before being euthanized. Blood samples were collected at the time of sacrifice and allowed to clot for 30 min. Then samples were centrifuged for 90 s at 6,000 *g*. FITC-dextran concentration was determined using fluorospectrometry at an excitation wavelength of 488 nm and emission wavelength of 520 nm.

### Bacterial Culture

To evaluate bacterial penetration within the colon as determined on day 7 after DSS treatment, a 1-cm segment of distal colon was sectioned longitudinally and the luminal contents were rinsed off with sterile PBS. The tissue was weighed, homogenized, and serially diluted with sterile PBS. The diluted homogenates were plated in triplicate on Brain Heart Infusion (BHI) agar and Blood agar (Hopebio-Technology, Qingdao, Shandong, China) and incubated for 24 h at 37°C. Colony-forming units (CFUs) were normalized to grams of intestinal tissue (CFU/g) to represent gut-associated bacterial counts (26).

### Ussing Chamber Studies

Mice were euthanized on day 3 after the DSS treatment. Colon mucosal membranes were harvested and mounted in Ussing chambers as previously described (27). Two-centimeter segments of the proximal colon were harvested and sectioned longitudinally along the mesenteric border. The seromusculature layers were removed by blunt dissection under a stereomicroscope. The mucosal membranes were then mounted between the two halves of the Ussing chamber with 0.28 cm^2^ of exposed area and each side of the tissue was bathed with 1 ml of Krebs solution at 37°C and saturated with CO_2_/O_2_ (5/95%). Tissues were voltage clamped to zero using an automatic voltage clamp and the transepithelial electrical resistance (TEER) was recorded.

### Alcian Blue-Periodic Acid Sthiff’s (AB-PAS) Staining

Mice treated with or without DSS were euthanized on day 3 after the GABA treatment. For the detection of acidic mucin, AB-PAS staining of sections from paraffin-embedded tissue were evaluated using a commercial AB-PAS kit (Solarbio Life Sciences, Beijing, China) according to the manufacturer’s instructions. Sections were stained by alcian blue solution (pH 2.5) for 15 min, washed in water for 5 min, then oxidized in 1% periodic acid at room temperature for 10 min, washed in water for 5 min, and stained by Schiff’s solution for 10 min.

### Immunohistochemistry and Immunofluorescence

Sections (4-μm thick) from paraffin-embedded colon tissue were prepared for immunohistochemistry or immunofluorescent staining. Briefly, staining was achieved with use of primary antibodies against GABA_A_ R β2/3 (1:200, mouse monoclonal, 05-474; Millipore), GAD 65/67 (1:100, mouse monoclonal, sc-365180; Santa Cruz), CD-45 (1:300, mouse monoclonal, 610265; BD Biosciences), mucin-2 (1:300; rabbit polyclonal, sc-15334; Santa Cruz), ki-67 (1:300; rabbit monoclonal, 12202S; CST), cleaved caspase-3 (1:300; rabbit polyclonal, 9661S; CST), junctional adhesion molecule 1 (JAM-1) (1:200; rabbit polyclonal, ab180821; Abcam), and occludin (1:200; rabbit polyclonal, ab168986; Abcam). As secondary antibodies, Alexa 488 conjugated-goat anti-rabbit antibody (1:1,000; A11008; ThermoFisher) or Alexa 568 conjugated-goat anti-mouse antibody (1:1,000; A-21043; Invitrogen) was used for immunofluorescent staining, while Broad Spectrum Zymed Poly HRP conjugated secondary antibodies (1:50; GK600505; Gene Tech) were used for immunohistochemistry. Human Caco-2 cells were fixed with 4% paraformaldehyde in 0.1 M phosphate buffer for 15 min. JAM-1 antibodies (1:200; rabbit polyclonal, ab180821; Abcam) were incubated overnight at 4°C. After three washes with PBS, slides were incubated with Alexa 568 conjugated donkey anti-rabbit antibody (1:1,000; A10042, Invitrogen) for 30 min and DAPI-containing PBST for 5 min.

### Protein Extraction and Western Blotting

Cells or colon tissues from each group were thawed and homogenized. The supernatants were collected for a protein assay. Protein extracts were isolated, separated by SDS PAGE, and transferred to a PVDF membrane (Millipore, Burlington, MA, USA). The membrane was incubated overnight with specific primary antibodies at 4°C. The membranes were incubated with chemiluminescent solution (Millipore, Burlington, MA, USA). Band density was determined using the Image J analyzer software (version 1.48).

### Electron Microscopy and Morphometry

At times indicated after DSS treatment, mice were euthanized, and 1-mm segments of distal colon were carefully dissected and fixed in 2.5% glutaraldehyde (Sigma-Aldrich, St. Louis, MO, USA) at 4°C. Samples were post-fixed with 1% OsO_4_ (Sigma-Aldrich, St. Louis, MO, USA) for 1 h, dehydrated, and embedded in resin. Ultra-thin sections (70-nm thick) were cut using an ultramicrotome (ultracut N; Leica, Germany) and collected on 300 mesh thin-bar copper grids. The sections were stained with uranyl acetate and lead citrate and were then observed using transmission electron microscopy (Philips Tecnai 20 U-Twin, Holland) and photographed.

### Statistics

All data represent the mean ± SEM unless otherwise indicated. To compare two groups, an unpaired *t*-test was used. To compare three or more groups, an ANOVA was performed followed by Student–Newman–Keuls’s *post hoc* test for pairwise comparisons. For data that failed to be normally distributed, the nonparametric Kruskal–Wallis test was used followed by the Mann–Whitney *U*-test for pairwise *post hoc* comparisons. A *P* value less than 0.05 was required for results to be considered statistically significant.

## Results

### GABAergic Signals Are Upregulated in Colon Mucosa of Colitis

**Figure 1:**
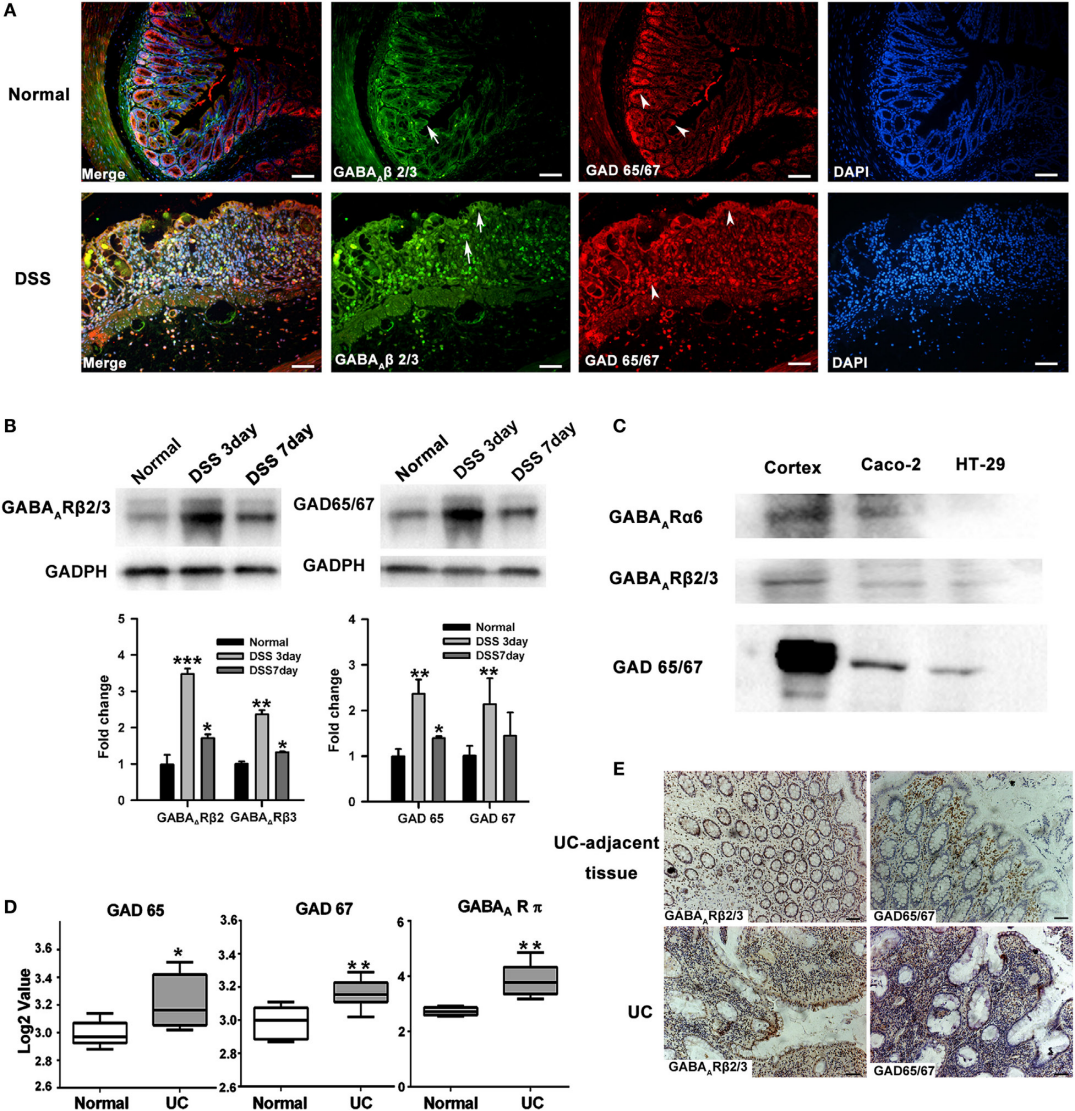
The GABAergic system is upregulated within the colon mucosa of ulcerative colitis (UC). **(A)** Representative immunohistochemical staining for the expression of GABA_A_ receptor (GABA_A_ R) β 2/3 and glutamic acid decarboxylase (GAD) 65/67 in colon mucosa of normal (upper panel) and DSS-exposed mice (lower panel) as determined on day 7 following DSS treatment (*n* = 8 per group). Arrow: GABA_A_ R β 2/3; arrow head: GAD 65/67. Scale bar 50 μm. **(B)** Western blot analysis of the expression of GABA_A_ R β 2/3 and GAD 65/67 in colon mucosa in normal and DSS-induced colitis mice as determined on day 7 following DSS treatment (*n* = 4 per group). **(C)** Western blot analysis of the expression of GABA_A_ Rs and GAD 65/67 in the human intestinal epithelial cells, Caco-2 and HT-29. **(D)** GAD 65, GAD 67, and GABA_A_ R π subunit expression levels in healthy controls and UC patients (using dataset GSE9452; normal: *n* = 5; UC: *n* = 8). **(E)** Representative immunohistochemical staining for the expression of GABA_A_ R β 2/3 (brown) and GAD 65/67 (brown) in UC patients and human UC-adjacent tissue (*n* = 3 per group), scale bar 100 μm. *P < 0.05, **P < 0.01, ***P < 0.001 vs. normal group.

The expression of GABAergic signals within colon mucosa of mice was first examined by assessing GABA_A_ Rs and GAD 65/67 expression using immunofluorescence. We found that GABA_A_ R β2/3 was present in both colonic epithelial cells and some LP cells, which probably represented infiltrating immune cells. GAD 65/67 was mainly expressed in intestinal epithelium and crypt cells, especially in the base of the crypt. Colonic GABA_A_ R and GAD 65/67 expressions were increased after DSS treatment (Figure 1A). Western blot results corroborated these findings (Figure 1B). In addition, we also found that expressions of GAD 65/67, GABA_A_ R α6, and β2/3 subunits were present in the human intestinal epithelial cell lines, such as Caco-2 and HT29 (Figure 1C). To investigate their expression in human colonic mucosa, we further analyzed GABAergic signals expression in the public gene dataset (dataset GSE9452) of UC samples. The results revealed that GAD 65, GAD 67, and the π subunit of GABA_A_ Rs were all upregulated in these samples when compared with those of samples from healthy controls (Figure 1D). Immunostaining also revealed high levels of GAD 65/67 and GABA_A_ Rs β2/3 subunits expressions were found in UC tissues when compared with UC-adjacent tissues (Figure 1E). Taken together, these results suggest that the upregulation of GABAergic signals in colorectal mucosa epithelium may be associated with the pathogenesis of UC.

### GABA Promotes DSS-Induced Colitis in Mice

**Figure 2:**
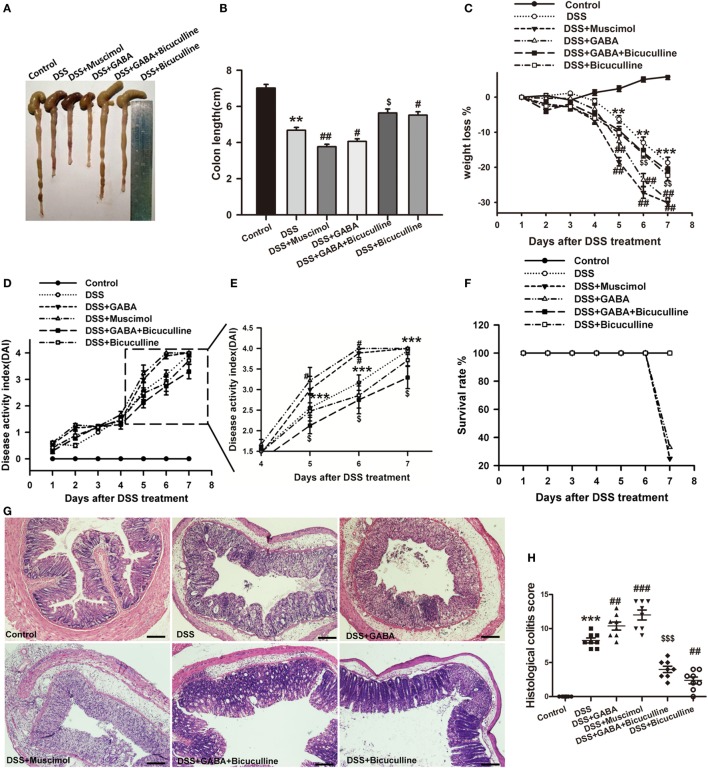
Gamma-aminobutyric acid (GABA) promotes DSS-induced acute colitis in mice. Mice receiving a 3% solution of DSS in their drinking water (*ad libitum*) were treated with or without GABA, a GABA_A_ receptor agonist or an antagonist and euthanized on day 7 after DSS treatment. **(A)** Representative photograph of the colon from each group. **(B)** The colon length was measured when mice were euthanized and average colon lengths were calculated (*n* = 8 per group). **(C)** Body weights were recorded daily during the experimental period (*n* = 8 per group). **(D)** Disease activity index (DAI) was estimated once per day for 7 days (*n* = 8 per group). **(E)** Magnified images of boxed regions as shown for DAI in panel **(D)**. **(F)** Survival rates were recorded daily for 7 days (*n* = 8 per group). **(G)** Representative portion of colon tissue stained by H&E, scale bar 200 μm. **(H)** Histological scores as estimated within each of the different groups (*n* = 8 per group). Values are the mean ± SE (*n* = 8); *P < 0.05, **P < 0.01, ***P < 0.001 vs. control group; ^#^*P* < 0.05, ^##^*P* < 0.01, ^###^*P* < 0.001 vs. the DSS group; ^$^*P* < 0.05, ^$$^*P* < 0.01, ^$$$^*P* < 0.001 vs. the DSS + GABA group.

**Figure 3:**
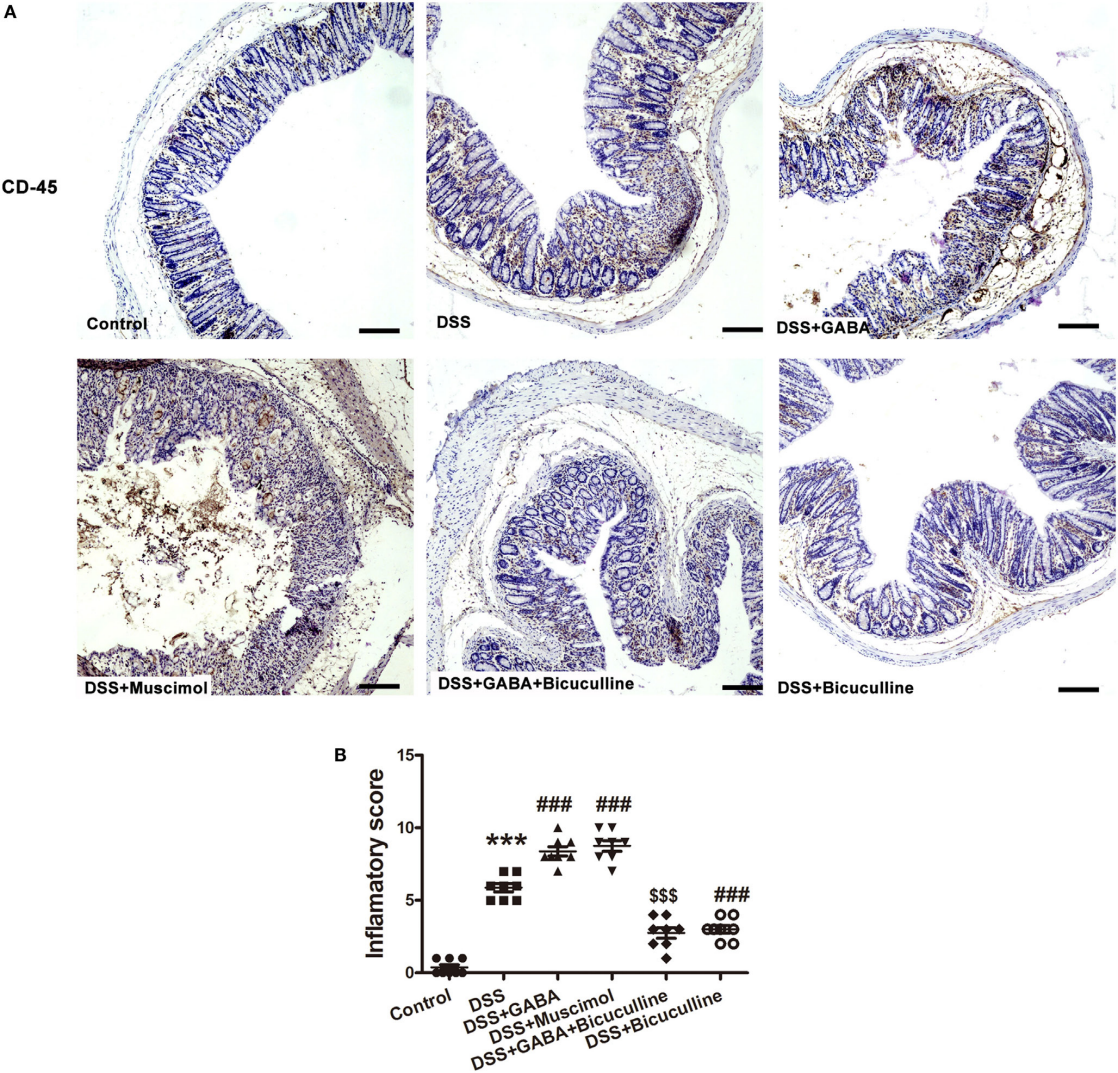
Gamma-aminobutyric acid (GABA) promotes leukocyte infiltration in DSS-induced colitis in mice. **(A)** Representative immunohistochemical data of CD-45 staining in colon mucosa of mice with or without GABA treatment. Leukocyte infiltration (brown) in the distal colon mucosa increased after DSS treatment compared with the control group, and further increment in leukocyte infiltration were obtained in response to GABA or muscimol co-treatment. These changes were reduced by treatment with bicuculline, scale bar 100 μm. **(B)** Bar graphs depicting inflammatory severity within the different groups (*n* = 8 per group). ***P < 0.001 vs. control group; ^###^*P* < 0.001 vs. the DSS group; ^$$$^*P* < 0.001 vs. the DSS + GABA group.

To determine the potential role of GABA in colonic inflammation, the induction of acute colitis was assessed in DSS mice treated (DSS + GABA) or not (DSS) with GABA. Our results showed that the DSS + GABA group had more severe colitis symptoms as indicated by significant reductions in colon length on day 7, greater losses of body weight and increases in the disease activity index (DAI) from day 5 after DSS treatment when compared with the DSS group (Figures 2A–E). Although no statistically significant differences were present for the DAI as determined on day 7, GABA reduced the survival rate to 33% in the DSS + GABA vs. 100% in the DSS group (Figure 2F). Histopathological analysis confirmed that DSS-induced colitis in mice treated with GABA showed more damage to their epithelium (Figures 2G,H) and substantially higher levels of leukocyte infiltration into their distal colon mucosa when compared with the DSS group (Figure 3). Further result showed that GABA_A_ Rs agonist, muscimol, also resulted in much more severe symptoms of colitis as GABA when compared with the DSS alone group. These changes were reduced by treatment with the GABA_A_ R competitive antagonist, bicuculline. To further evaluate the effect of endogenous GABA in this mouse colitis model, co-administration of bicuculline to DSS group significantly reduced colitis symptoms and epithelial damage. These results suggest that both endogenous and exogenous GABA exacerbate DSS-induced acute colitis through activation of GABA_A_ Rs.

**Figure 4:**
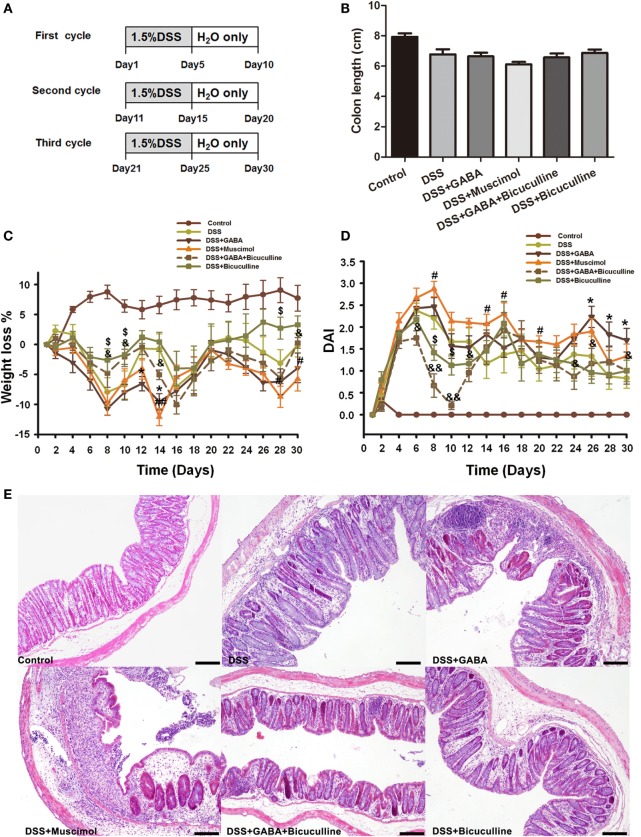
Gamma-aminobutyric acid (GABA) promotes DSS-induced chronic colitis in mice. **(A)** Experimental scheme of DSS-induced chronic colitis model. Mice received 1.5% DSS in the drinking water for 5 days followed by a recovery phase with normal water for 5 days. This cycle was repeated thrice. Mice of the control group received normal water over the whole period of time. **(B)** The colon length was measured when mice were euthanized and average colon lengths were calculated (*n* = 8 per group). **(C)** Body weights were recorded daily during the experimental period. **(D)** Disease activity index (DAI) was estimated once per day during the experimental period. **(E)** Representative portion of colon tissue stained by H&E, scale bar 100 μm. Values are the mean ± SE (*n* = 8); asterisks represent statistically significant differences between DSS + GABA and the DSS group; *P < 0.05; pound signs represent statistically significant differences between DSS + Muscimol and the DSS group; ^#^*P* < 0.05, ^##^*P* < 0.01; symbol $ represents statistically significant differences between DSS + Bicuculline and DSS group; ^$^*P* < 0.05; symbol & represents statistically significant differences between DSS + GABA + Bicuculline and DSS + GABA group; ^&^*P* < 0.05, ^&&^*P* < 0.01.

To further determine the potential role of GABA in chronic colitis, we assessed the body weight loss, DAI, colon length, and histological damage in different groups as indicated. Our results showed although there was no significant difference in colon length in different groups (Figure 4B), chronic DSS group showed increased weight loss and DAI score when compared with the control group at each time point. GABA increased weight loss on days 12 and 14 and muscimol increased weight loss on days 14, 28, and 30 when compared with the DSS alone group. GABA increased the DAI on days 26, 28, and 30 and muscimol increased the DAI on days 8, 14, and 16 when compared with the DSS alone group. Bicuculline partly blocks these changes (Figures 4C,D). Consistent with the results in the acute colitis model, histopathological analysis found that DSS-induced chronic colitis in mice treated with GABA or muscimol showed more histological damage to their epithelium. These histological damages were reduced by co-treatment with bicuculline (Figure 4E).

### GABA Inhibits Pro-Inflammatory Cytokine Production in DSS-Induced Colitis

**Figure 5:**
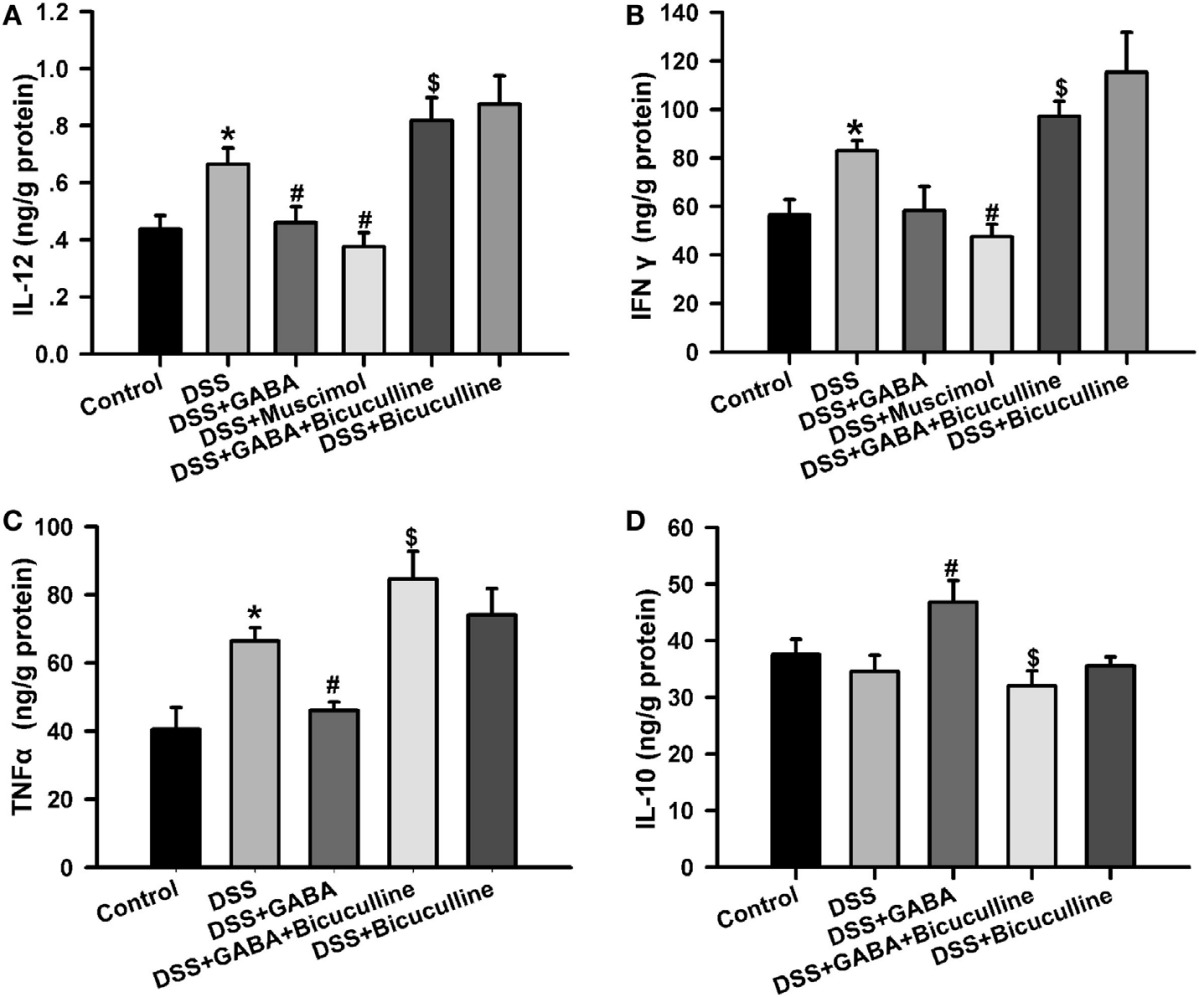
Gamma-aminobutyric acid (GABA) reduces pro-inflammatory cytokine production in DSS-induced colitis colon. **(A)** IL-12, **(B)** IFN-γ, **(C)** TNF-α, and **(D)** IL-10 levels within the different groups as determined using enzyme-linked immunosorbent assay kits. Values are the mean ± SE (*n* = 8 per group); *P < 0.05 vs. control group; ^#^*P* < 0.05 vs. the DSS group; ^$^*P* < 0.05 vs. the DSS + GABA group.

Enzyme-linked immunosorbent assay results showed that tissue IL-12, IFN-γ, and TNF-α level were significantly higher in the DSS treatment group when compared with that of the control group. These effects were significantly decreased by GABA or muscimol treatments while a co-administration of bicuculline reversed these changes. Furthermore, GABA significantly increased colon IL-10 production in the DSS + GABA group vs. that observed in the DSS alone group, again, an effect which was reversed with a co-administration of bicuculline (Figure 5). These data suggest that both endogenous and exogenous GABA significantly decreased pro-inflammatory cytokine production, but increased production of the anti-inflammatory cytokine, IL-10, through activation of GABA_A_ Rs. These results are consistent with previous studies (8, 14, 15). Blocking of GABA_A_ Rs was also shown to significantly increase the production of IL-12 and IFN-γ when compared with levels observed in the DSS group.

### GABA Increases the Permeability of Colon Mucosa in Colitis

**Figure 6:**
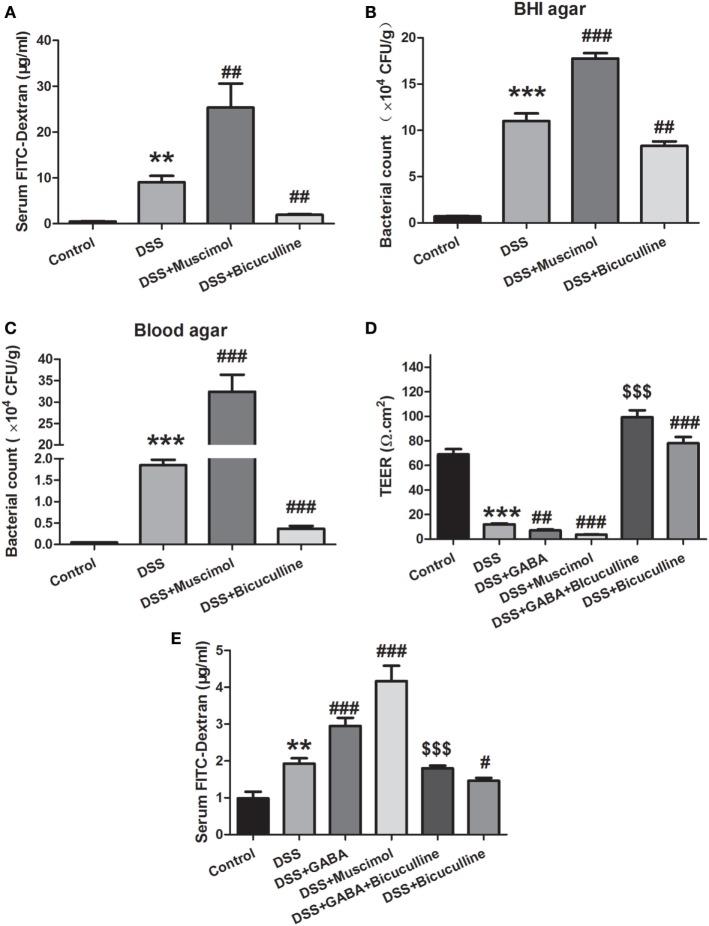
Gamma-aminobutyric acid (GABA) increases the permeability of colon mucosa in colitis. **(A)** Mice received an oral gavage of FITC-dextran (0.4 mg/g) and sera FITC-dextran concentrations were determined 4 h later (*n* = 8 per group) in acute colitis model. **(B)** Bacterial counts in colonic tissues were determined with a colony-forming assay using Brain Heart Infusion (BHI) agar (*n* = 6–8 per group). **(C)** Bacterial counts in colonic tissues were determined with a colony-forming assay using blood agar (*n* = 6–8 per group). **(D)** Transepithelial electrical resistance was measured within the different groups with use of the Ussing chamber (*n* = 6 per group). **(E)** Mice received an oral gavage of FITC-dextran (0.4 mg/g) and sera FITC-dextran concentrations were determined 4 h later (*n* = 8 per group) in chronic colitis model. Values are the mean ± SE; *P < 0.05, **P < 0.01, ***P < 0.001 vs. control group; ^#^*P* < 0.05, ^##^*P* < 0.01, ^###^*P* < 0.001 vs. the DSS group; ^$^*P* < 0.05, ^$$^*P* < 0.01, ^$$$^*P* < 0.001 vs. the DSS + GABA group.

An *in vivo* intestinal permeability assay was performed to assess barrier function using FITC-dextran on day 7 of DSS treatment. Serum FITC-dextran in DSS-induced colitis was greater than that observed in control mice and treatment with muscimol further increased levels of serum FITC-dextran, indicating more severe damage of intestinal barrier function. Bicuculline alleviated this increment in serum FITC-dextran in DSS-treated mice (Figure 6A). Consistently, the number of bacteria in colonic tissues was found to be increased in the DSS group as indicated in both BHI agar and blood agar. Treatment with muscimol further promoted these increments in penetrating bacterial numbers while bicuculline co-administration decreased bacterial numbers in the colon tissue in DSS-induced colitis (Figures 6B,C). These data indicate that GABA increased intestinal permeability and bacterial penetration through activation of GABA_A_ Rs.

Tissue TEER measurements serve as an indicator for epithelial barrier integrity. We found that DSS significantly reduced mucosal TEER compared with that observed within the control group as determined on day 3. Day 3 was selected for this analysis as mucosal damage was too severe to be harvested on day 7. Either GABA or muscimol produced additional decreases of TEER in this DSS-induced colitis model while bicuculline reversed these changes (Figure 6D). These data indicate that GABA aggravated intestinal barrier damage by affecting the integrity of the epithelial barrier.

To investigate the effect of GABA on intestinal permeability in chronic colitis, mice were also gavaged with FITC-dextran at 4 h before being euthanized. Consistent with the results in the acute colitis model, chronic DSS treatment significantly increased serum FITC-dextran concentration when compared with the control group. Treatment with GABA or muscimol further increased levels of serum FITC-dextran when compared with the DSS-induced chronic colitis group. These increments were reduced by treatment with bicuculline (Figure 6E).

### Detection and Observation of Intestinal Mucosal Ultrastructure

**Figure 7:**
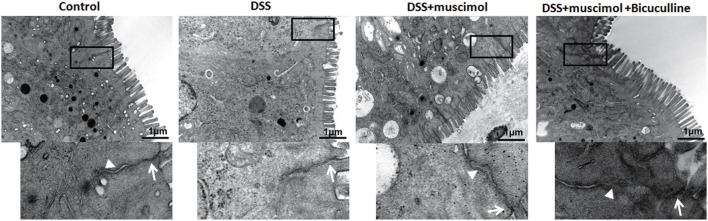
Transmission electron micrographs of tight junction (TJ) regions in colon enterocytes. Lower panel: magnified image of boxed regions; arrow: TJs; and arrow head: adherens junction (*n* = 4 per group), scale bar 1 μm.

Destruction of TJs is a major factor contributing to epithelial dysfunction. In our experiments, an enlarged TJ and expansion of the endoplasmic reticulum were observed in both the DSS and DSS + GABA groups when compared with that of the control group on day 7. Adheren junctions were expanded and more phagocytotic vesicles were found in the DSS + GABA vs. DSS group, indicating that more severe injuries were induced by the presence of GABA. The damage resulting from GABA was significantly ameliorated following bicuculline treatment (Figure 7).

### GABA Decreased the Expression of Junctional Adhesion Molecule (JAM) and Occludin Between Colonic Epithelial Cells

**Figure 8:**
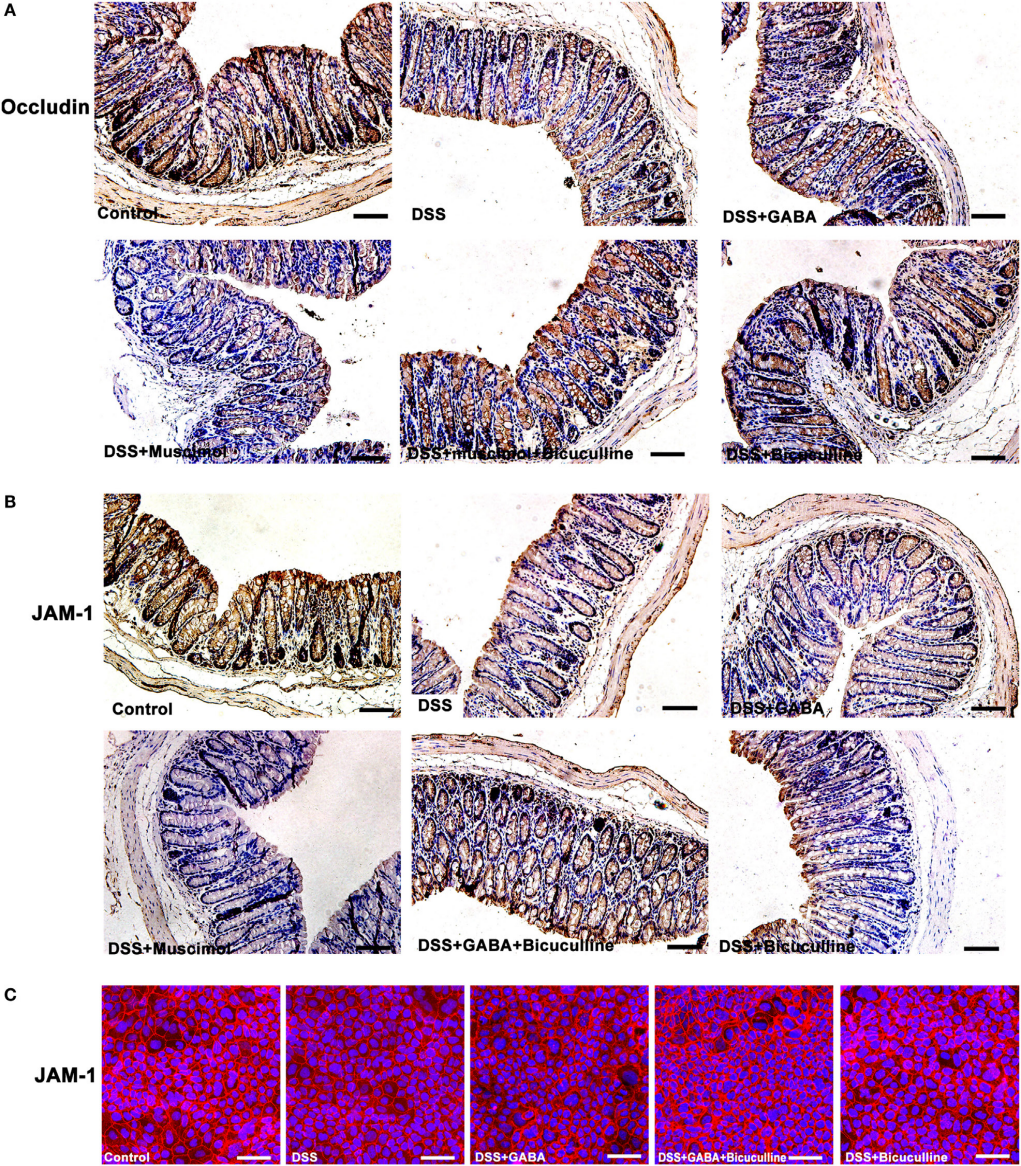
Gamma-aminobutyric acid (GABA) reduces the expression of occludin and junctional adhesion molecule 1 (JAM-1) in mice. **(A)** Representative immunohistochemical data for occludin staining (brown) in colon mucosa within the different groups, scale bar 100 μm. **(B)** Representative immunohistochemical data for JAM-1 staining (brown) in colon mucosa within the different groups, scale bar 100 μm. **(C)** Representative immunofluorescence staining of JAM-1 (red) in DSS-treated Caco-2 cells with or without GABA, scale bar 25 μm.

Tight junction protein complexes include JAMs, occludin (28), and claudins (29). JAMs and occludin play central roles in the physical regulation of paracellular permeability (30). Our results showed that GABA and muscimol decreased JAM-1 and occludin expression on day 3 in the DSS-induced colitis mouse model, an effect which was blocked by bicuculline (Figures 8A,B). Similar results were obtained in our *in vitro* study using Caco-2 cells (Figure 8C). These data indicate that decrements in JAM-1 and occludin expression may be responsible for the decrease in mucosal TEER.

### GABA Disrupted the Mucus Barrier

**Figure 9:**
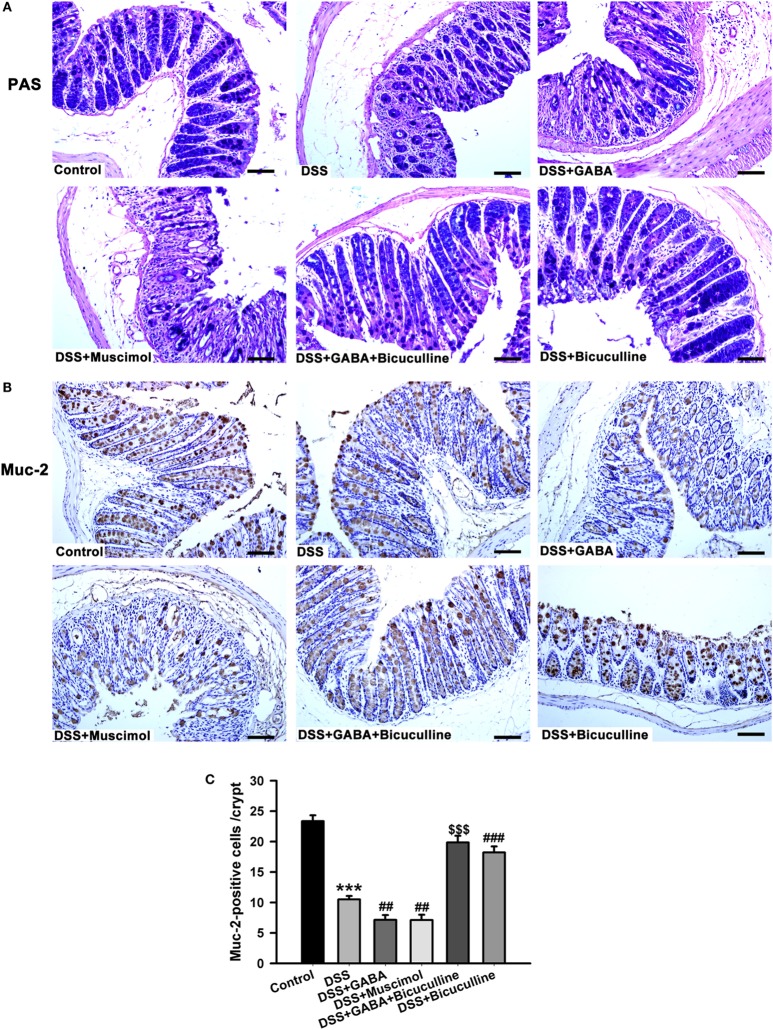
Gamma-aminobutyric acid (GABA) inhibits mucous secretion. Mice receiving a 3% solution of DSS in their drinking water (*ad libitum*) were treated with or without GABA, a GABA_A_ receptor agonist or an antagonist and euthanized on day 3. **(A)** Representative staining of mucous layers (blue) within the different groups, scale bar 50 μm. **(B)** Representative immunohistochemical staining of muc-2 (brown) in colon mucosa within the different groups, scale bar 50 μm. **(C)** Bar graphs depicting the number of muc-2-positive cells in one crypt within the different groups. ***P < 0.001 vs. control group; ^##^*P* < 0.01, ^###^*P* < 0.001 vs. the DSS group; ^$$$^*P* < 0.001 vs. the DSS + GABA group.

We next assessed mucous layers in all groups on day 3 following DSS treatment. A significant reduction in the mucous layer was observed in DSS-induced colitis mice treated with either GABA or muscimol, an effect which was blocked by bicuculline (Figure 9A). Since mucus within the colon is mainly secreted by goblet cells, we next examined whether GABA affected the goblet cells as determined by evaluating muc-2 expression. Our results showed that goblet cell numbers per crypt decreased in GABA treated DSS-induced colitis colon. Bicuculline blocked the reduction induced by exogenous and endogenous GABA (Figures 9B,C). These data indicate that activation of GABA_A_ Rs decreased mucous layer thickness *via* reductions in goblet cell numbers.

### GABA Inhibits Colonic Epithelial Cell Proliferation and Increases Its Apoptosis

**Figure 10:**
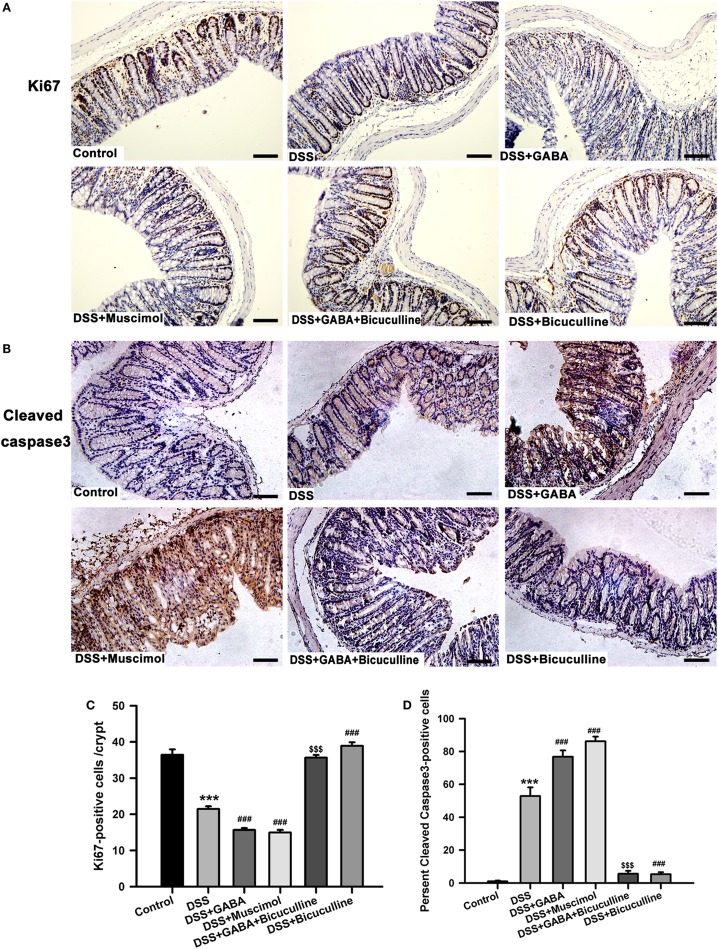
Gamma-aminobutyric acid (GABA) inhibits the proliferation and promotes apoptosis of colon enterocytes on day 3. Representative immunohistochemical staining of ki-67 (brown) **(A)** and cleaved caspase-3 (brown) **(B)** in colon mucosa within the different groups, scale bar 100 μm. Bar graphs depicting ki-67-positive cells **(C)** and cleaved caspase-3-positive cells **(D)** in one crypt within the different groups. Values are the mean ± SE (*n* = 8 per group). ***P < 0.001 vs. control group; ^###^*P* < 0.001 vs. the DSS group; ^$$$^*P* < 0.001 vs. the DSS + GABA group.

Immunohistochemistry results showed that the number of ki-67-positive cells decreased significantly after 3 days following DSS treatment when compared with that of the control group. GABA or muscimol administration to DSS mice further reduced the number of ki-67-positive cells in comparison with that of the DSS alone group (Figures 10A,C). Taken together, these data provide strong evidence indicating that reductions in ki-67-positive cells were a major factor contributing to crypt destruction. We next examined whether GABA affected colonic epithelial cell apoptosis in colitis mice. Results showed either GABA or muscimol further increased the number of cleaved caspase-3-positive cells in DSS-induced colitis, while bicuculline blocked these changes (Figures 10B,D). These data indicate that activation of GABA_A_ Rs induces apoptosis of epithelial cells in colitis colon.

## Discussion

Gamma-aminobutyric acid, through its major inhibitory role within the CNS, regulates neuronal excitability. However, recent data demonstrate that GABA is also critical in ameliorating immune and inflammatory responses as demonstrated in several autoimmune diseases. Our present results reveal that GABAergic signals are upregulated and associated with the pathogenesis of UC in colon tissue as demonstrated in both UC patients and in the DSS-induced mouse colitis model. Although GABA significantly decreased pro-inflammatory cytokine levels and increased anti-inflammatory cytokine production in DSS-induced acute colitis, surprising, unlike that in autoimmune diseases, GABA accelerated the onset and progression of DSS-induced acute colitis, an effect which was blocked by bicuculline. Further results showed that GABA inhibited mucosal epithelia reproduction and promoted its apoptosis in colitis colon. Such an effect produces a disruption in intestinal barrier integrity and a reduction in goblet cells, eventually leading to a reduction of the mucous layer. These findings suggest that GABA disrupted intestinal barrier, increased bacterial penetration which enhances immune and inflammatory damage to the colon mucosa.

Our current findings show that β2/3 subunits of GABA_A_ R and GAD 65/67 were present in epithelial cells and some LP cells in both mouse and human colon. And, results from Western blots revealed that the subunits α5 and β2/3 of GABA_A_ R and GAD 65/67 were also expressed in human colorectal epithelial cells. Although we did not detect other subunits of GABA_A_ Rs, these data indicate that GABAergic signaling exists in the colon mucosa and that GABA can be synthesized locally and may function as a paracrine or autocrine factor. Moreover, our results from analysis of the public gene datasets of UC samples showed an upregulation of GABA_A_ Rs π subunit and GAD 65/67 in colorectal mucosa epithelium of UC patients. The immunohistochemistry and immunofluorescence results obtained from our colonic biopsy specimens of UC patients as well as the DSS-induced mouse colitis model further corroborated these results. Therefore, these results suggest that there is more GABA production and GABA_A_ R expression within the colon mucosa of UC patients, and the upregulation of GABAergic signals might be associated with the pathogenesis of UC. To determine the potential role of GABA in colonic inflammation, colon lengths, body weights, survival rate, DAI, and a histological analysis were performed and compared between DSS + GABA and DSS groups. We found that DSS-induced colitis in mice treated with GABA or muscimol showed more severe damage to colon epithelium and substantially higher levels of leukocyte infiltration into their distal colon mucosa when compared with the DSS group. These changes were reduced by treatment with the GABA_A_ R competitive antagonist, bicuculline, indicating that GABA promoting DSS-induced colitis through activation of GABA_A_ Rs. However, these findings would seem to be contrary to that of the well-characterized function of GABA, which has been reported to inhibit inflammation and ameliorate autoimmune diseases (31). Therefore, we next investigated whether GABA exerts the same suppressive effects upon cytokine secretion in colitis as that observed in autoimmune diseases. Our results showed that GABA administration reduced colon levels of inflammatory cytokines such as TNF-α, IFNγ, and IL-12 in DSS-induced colitis mice, while significantly increasing tissue levels of IL-10. These data are consistent with those changes observed in autoimmune diseases. Thus, GABA yields totally opposite effects on immune and inflammatory damage in colitis vs. autoimmune diseases, although it induced the same suppression effect on pro-inflammatory cytokines. The possible reasons will be discussed later. Based on these results, we speculated that the accelerating inflammatory damage induced by GABA in our model was not mediated by inflammatory cytokine expression.

Intestinal barrier dysfunction plays a key role in the initiation and propagation of UC. Therefore, we examined whether GABA exacerbated colitis through contributing to intestinal barrier defects. Our results showed treatment with muscimol resulted in an increment of serum FITC-dextran and bacterial invasion in colonic tissues when compared with that of the DSS alone group, indicating that it increased the permeability of the intestinal barrier. Bicuculline prevented this increase in permeability and the resultant increase in bacterial numbers within the colon tissue of colitis. Therefore, our data suggest that GABA increased intestinal permeability and bacterial penetration within the colon through activation of GABA_A_ Rs.

The intestinal barrier includes both an epithelial and mucosal barrier. In our study, GABA reduced TEER, indicating that GABA aggravated the damaged intestinal barrier by affecting the epithelial integrity. Ultrastructure results also confirmed that tight junctional damage and more severe cellular injuries were induced by GABA. Bicuculline blocked these changes. Further study of this issue indicated that GABA and muscimol reduced JAM-1 and occludin expression in colitis mice. And, results from our *in vitro* study showed that GABA decreased JAM-1 staining in DSS-treated Caco-2 cells, which suggest one potential reason for the decrement of mucosa TEER. Treatment with bicuculline blocked these changes as determined. These data provide further evidence indicating that GABA also inhibits the expression of barrier-forming components of TJs through GABA_A_ Rs. However, whether GABA_A_ Rs activation has a direct or an indirect impact on TJs and the underlying mechanisms are still not known. This is clearly a case where further work is needed.

The intestinal mucosa is enclosed by a thick mucus layer, which serves as a major barrier between the epithelium and lumen of the host GI tract and plays an essential role in maintain homeostasis of the intestinal mucosa (32). Our present results reveal that GABA or muscimol significantly reduced this mucous layer in DSS-induced colitis mice, effects which were blocked by bicuculline. In addition, this reduction in the mucosal layer as induced by GABA was shown to be due to a reduction in goblet cells. Therefore, we conclude that GABA disrupted the mucous barrier through reducing goblet cell numbers via activation of its GABA_A_ Rs.

To elucidate the possible mechanisms of goblet cells reduction induced by GABA, we further investigated the effects of GABA on epithelia proliferation and apoptosis in colitis. We found that GABA and muscimol significantly reduced the number of ki-67-positive cells and increased cleaved caspase-3-positive cells in the colon when compared with that of the DSS alone group, effects which were blocked by bicuculline. These results indicate that GABA and muscimol decrease the formation of goblet cells, most likely by inhibiting epithelia proliferation and/or inducing apoptosis of colonic epithelial cells in colitis. In addition, the increased apoptosis of epithelial cells might also be partly responsible for the aforementioned destruction of TJ induced by GABA_A_ Rs activation.

As discussed previously, GABA reduced the increments of pro-inflammatory cytokines and increased anti-inflammatory cytokines in the colitis colon. Based on these changes in cytokines within the colon, it was anticipated that mucosal inflammatory reactions should be inhibited by GABA treatment. On the contrary, GABA promoted histological damage in this DSS-induced mouse model of colitis. Traditionally, it is well known that inflammation underlies many chronic and degenerative diseases and chronic unresolved inflammation culminates in a host of pathologies such as cancer and fibrosis. However, inflammation also serves an important protective function that can eliminate its primary initiates such as foreign organisms, dead cells, or physical irritants. It has been reported that inflammatory cytokines, including TNF-α, IL-4, and IL-13, promote mucin gene expression in intestinal epithelial cells and prevent pathogen infection and colonization (33, 34). A recent paper also demonstrated that anti-TNF-α therapy in inflammatory bowel disease (IBD) induced a twofold increase in the risk of opportunistic infections (35). Inflammation also plays a crucial role in the regeneration of injured tissues (34). In this way, insufficient inflammatory responses can result in tissue destruction by harmful initiates, especially bacteria (36). In the gastrointestinal tract, the large intestine harbors the highest numbers (10^13^–10^14^) of commensal bacteria (37, 38). Under normal conditions (non-colitic colon), infiltrated immune and inflammatory cells may have the capacity to produce sufficient amounts of cytokines to eliminate the bacteria and inhibit the resultant severe tissue damage. However, in our model, GABA suppressed tissue pro-inflammatory cytokines and increased anti-inflammatory cytokine expression. Thus, colon bacteria invasion would not be effectively eliminated, thereby partly contributing to the histological damage observed. In this way, on the one hand, GABA induced destruction of the gut barrier and increased bacterial penetration, and on the other hand, it reduced tissue pro-inflammatory cytokines in colon mucosa which led to the reduced elimination of invasive bacteria. Thus, although GABA application in UC results in a decrease of cytokine secretion, like that as seen in many autoimmune diseases without bacterial invasion, it can also contribute to the destruction, rather than the recovery of colon tissue under colitis conditions.

In summary, we found an upregulation of GABAergic signaling in UC which contributed to damage of intestinal barrier function and accelerated the histological damage. However, a previous study indicated that oral administration of topiramateis able to significantly ameliorate an IBD phenotype in a trinitrobenzenesulfonic acid (TNBS)-induced rodent model of colitis (39). Although topiramate is known to enhance GABAergic neurotransmission, it also has a broad spectrum of action such as blocking AMPA and kainate receptors, and sodium and calcium channels as well as inhibiting carbonic anhydrase (40, 41). Thus, it is hard to rule out the possibility that other factors contribute to the therapeutic effects of topiramate to TNBS-induced colitis. Additional studies are needed to determine the precise mechanism of topiramate in colitis. Work by Aggarwal et al. (42) recently reported that GABA level decreased in serum and colon biopsy of UC patients, although GABA_A_ R π subunit increased significantly. The authors speculated that reduced GABA level contributed to pathogenesis of UC. One explanation for the discrepancy may be due to different models or different clinical course of the disease. In addition, as the authors had not studied the effect of blocking GABAergic signal in UC, extreme caution should be exercised when drawing the conclusion of GABA reduction as a contributory factor for UC. Thus, further scientific efforts are needed to clarify the specific contribution of GABAergic system in UC.

Although acute DSS colitis is a good model to study the inflammatory onset caused by epithelial barrier destruction, it occurs in the absence of mature lymphocytes. Therefore, we performed an initial pilot validation experiment using DSS-induced chronic colitis model to determine whether GABA_A_ Rs activation plays the same role in chronic intestinal inflammation. Consistent with its role in acute colitis, GABA, or muscimol treatment also leads to more severe epithelial damage and an increment of serum FITC-dextran when compared with DSS alone group, suggesting that GABA_A_ Rs activation increased the intestinal permeability in chronic colitis. Co-administration of bicuculline significantly reduced epithelial damage and the increment of intestinal permeability. Therefore, these results suggest that GABA_A_ Rs activation also facilitates the development of chronic colitis. However, further studies are needed to investigate the roles and mechanisms using different colitis models.

Recently, GABA has attracted a considerable amount of attention for its potential use as a functional food. Accumulating evidence indicates that GABA supplemented foods have beneficial effects, such as improvements in memory, reductions in blood pressure, and anti-anxiety effects (43–45). It has even been authenticated as a safe food supplement in a number of countries, such as US, China, and Japan. The dose limit for use of GABA in China is 500 mg/day. However, our current findings found that only 5 mg/kg of GABA as administration i.p. dramatically disrupted the mucosal barrier in DSS-induced colitis mouse model, and affected the vulnerability of colon to the induction of inflammatory processes. These findings suggest that GABA may have a potential role in inducing or exacerbating inflammatory damage within the colon. Clinically, GABA_A_ R agonists are widely used such as benzodiazepines, barbiturates, propofol, and isoflurane (46). Accordingly, we suggest that caution should be exercised with regard to use of GABA supplements or drugs acting on GABA_A_ Rs, especially in patients with gastrointestinal disease. Further clinical studies are needed to elucidate the role of GABA in mucosal barrier function, especially in patients treated with GABAergic drugs.

## Ethics Statement

Ethical approval for the use of archival tissue was provided by the Medical Ethics Committees of Shandong University (number MECSDUMS 2010032). Informed consent was obtained from each patient. All animal procedures were approved by the Medical Ethics Committee for Experimental Animals, Shandong University (number LL-201602017).

## Author Contributions

XM and JL designed the experiments, analyzed and interpreted data, and wrote the manuscript. XM and QS performed the experiments and provided technical support. XS, CW, DC, and XY performed some experiments; CL and YL helped analyze data and provided scientific advice.

## Conflict of Interest Statement

The authors declare that the research was conducted in the absence of any commercial or financial relationships that could be construed as a potential conflict of interest.
